# Machine learning for infection risk prediction in postoperative patients with non-mechanical ventilation and intravenous neurotargeted drugs

**DOI:** 10.3389/fneur.2022.942023

**Published:** 2022-08-01

**Authors:** Yi Du, Haipeng Shi, Xiaojing Yang, Weidong Wu

**Affiliations:** Department of Intensive Care Medicine, Shanxi Bethune Hospital, Shanxi Academy of Medical Sciences, Taiyuan, China

**Keywords:** machine learning, post-surgical ICU, neurovascular, anesthesia, infection, hydromorphone

## Abstract

Drug efficacy can be improved by understanding the effects of anesthesia on the neurovascular system. In this study, we used machine learning algorithms to predict the risk of infection in postoperative intensive care unit (ICU) patients who are on non-mechanical ventilation and are receiving hydromorphone analgesia. In this retrospective study, 130 patients were divided into high and low dose groups of hydromorphone analgesic pump patients admitted after surgery. The white blood cells (WBC) count and incidence rate of infection was significantly higher in the high hydromorphone dosage group compared to the low hydromorphone dosage groups (*p* < 0.05). Furthermore, significant differences in age (*P* = 0.006), body mass index (BMI) (*P* = 0.001), WBC count (*P* = 0.019), C-reactive protein (CRP) (*P* = 0.038), hydromorphone dosage (*P* = 0.014), and biological sex (*P* = 0.024) were seen between the infected and non-infected groups. The infected group also had a longer hospital stay and an extended stay in the intensive care unit compared to the non-infected group. We identified important risk factors for the development of postoperative infections by using machine learning algorithms, including hydromorphone dosage, age, biological sex, BMI, and WBC count. Logistic regression analysis was applied to incorporate these variables to construct infection prediction models and nomograms. The area under curves (AUC) of the model were 0.835, 0.747, and 0.818 in the training group, validation group, and overall pairwise column group, respectively. Therefore, we determined that hydromorphone dosage, age, biological sex, BMI, WBC count, and CRP are significant risk factors in developing postoperative infections.

## Introduction

Patients in the intensive care unit (ICU) experience varying degrees of pain, delirium, agitation, and arousal. These conditions can lead to prolonged ICU stays and increased morbidity and mortality (1–4). As a result, analgesia and sedation have become part of routine ICU care, leading to the current e-CASH (early comfort using analgesia, minimal sedatives, and maximal humane care) sedation concept (5). The development of clinical drugs used for analgesia and sedation of ICU patients has been increasing in number. Assessing the condition and determining its pharmacology can lead to better analgesia with narcotics (6–9). In the ICU, we are more likely to make better treatment decisions if we understand the different effects of anesthesia on the neurovascular system (10).

Morphine is widely used internationally as a classical neurovascular anesthetic because of its low price and significant effects (11–13). However, repeated administration of morphine can result in serious adverse events (14). Hydromorphone, a new analgesic drug, is a semi-synthetic morphine derivative. The analgesic effect is approximately 8–10 times better than morphine, and it has fast onset of action, fewer side effects, various routes of administration, low risk of hallucination addiction, low risk of gastrointestinal adverse reactions, non-toxic metabolites, and better suitability for patients with decompensated liver and kidney function (13, 15). Hydromorphone has been extensively validated for use in an emergency, day surgery, and perioperative management settings, with significant advantages in reducing respiratory depression and gastrointestinal adverse effects (15–18). However, further studies are needed to assess the effectiveness, safety, and dosage of this medication in the ICU.

In contrast to other analgesics, hydromorphone has been found to reduce the incidence of postoperative pneumonia in patients undergoing thoracic surgery; however, in general, infection remains to be a significant complication of hydromorphone (17, 19, 20). For one, an increased incidence of infective endocarditis has been associated with hydromorphone injection (20). Similarly, intrathecal targeted drug delivery of hydromorphone may lead to device infection (21). These complications may be problematic since inflammation and infection have been shown to reduce the effectiveness of some analgesics (22). Moreover, the postoperative infection has been found to be an important risk factor affecting the survival of patients (23, 24). These suggest that in clinical practice, the risk of infection needs to be predicted.

Machine learning is currently an important means of implementing artificial intelligence technologies, and the application of these algorithms in clinical diagnosis and decision-making has become a research priority in the medical field (25–27). In non-mechanically ventilated ICU, predicting the risk of hydromorphone infection is very challenging and requires an accurate biological classification model utilizing simple and effective decision rules. In relation to this, random forest models and support vector machine models are frequently used in classification tasks to predict treatment effects or complications and to screen for clinically important features related to outcomes (28). Logistic regression analysis is often used to screen for risk factors associated with adverse outcomes, and it can also be utilized in constructing a nomogram that can be easily applied clinically for the accurate detection and treatment of a disease (29, 30). In non-mechanically ventilated ICU patients after surgery, we believe that these machine learning tools can be used to predict the risk of infection with hydromorphone analgesia.

The purpose of this study is to develop a validated tool that can utilize available clinical information to predict the risk of infection in ICU patients receiving post-surgical hydromorphone analgesia. This will ultimately reduce hospital stays, medical costs, and length of hospital stays post-operatively, as well as provide guidance for future studies to improve analgesic outcomes for ICU patients.

## Method

### Clinical cohort data acquisition

After institutional ethics committee approval, a group of patients admitted to the ICU after surgical procedures and who received analgesic treatment was enrolled in the study. Since this study used a retrospective analysis format, patients' informed consent was waived. The inclusion criteria used were as follows: (1) age between 45 and 90; (2) American Society of Anesthesiologists (ASA) physical score I to II; and (3) cardiac left ventricular ejection fraction greater than 40%. Meanwhile, the exclusion criteria used were as follows: (1) those with significant preoperative heart, liver, or kidney disease; (2) those with neurological or psychiatric diseases; (3) those with lung conditions such as chronic obstructive pulmonary disease or chronic bronchitis; (4) those with allergies to study drugs such as opioid prescriptions; and (5) those with a body mass index of 30 kg/m^2^. Overall, a total of 130 patients passed the screening criteria. The general characteristics of these patients, such as biological sex, age, height, weight, BMI, etc., were collected through a clinical history data review. In addition, Acute Physiology and Chronic Health Evaluation (APACHE II) and Sepsis Related Organ Failure Assessment (SOFA) scores were collected in full at the time of patient admission to the ICU.

### Hydromorphone dosage calculation

The pumping speed of the micropump was adjusted to achieve satisfactory analgesia. Patients were evaluated for pain and physiological indicators every 2 h. Patients whose daily hydromorphone dosage exceeded 40 mg/mL were included in the high hydromorphone dosage group, and those who did not exceed 40 mg/mL were included in the low hydromorphone dosage group.

### Closing indicators

Physiological and biochemical indices, such as heart rate (HR), mean artery pressure (MAP), respiratory rate (R), C-reactive protein (CRP), white blood cell (WBC) count, platelet (PLT), and Saturation of Pulse Oxygen (SpO2), were recorded completely after 12 h of analgesia, from the moment that the patient was admitted to ICU with self-administered hydromorphone analgesia. In addition, the occurrence of adverse reactions throughout the postoperative treatment period was recorded. In this study, the primary outcome indicator was defined as the occurrence of infection. Postoperative infection was evaluated based on the following indicators: (1) temperature >38 °C; (2) elevated WBC count; (3) positive sputum culture or blood culture showing bacteria; (4) chest X-ray showing abnormal density; and (5) diagnosis of pneumonia, etc. (31).

### Machine learning and logistic regression analysis

Firstly, a random forest model was utilized to fit this dataset so as to rank the importance of each clinical feature in terms of the infection outcome (32). Subsequently, in order to screen clinical features related to infection, Support Vector Machine-Recursive Feature Elimination (SVM-RFE) was applied to the general characteristics of patients, secondary indicators for the prediction model of infection important features screening and model construction (33). A support vector machine (SVM) is often used for non-linear classification, and they perform well on small samples. The SVM-RFE technique uses iterative iterations to remove redundant feature variables. Root Mean Squard Error (RMSE) metrics were applied to evaluate the accuracy of the SVM models and to determine the best model variables based on the RMSE minimum. Furthermore, neural networks, a common non-linear algorithmic model in machine learning, were also used to evaluate results as previous researches (34–38).

Univariate and multifactorial logistic regression analyses were also applied to assess the risk ratio of each clinical factor on infection outcome (39). In the first step, we performed univariate logistic regression analysis sequentially and analyzed the characteristics of the patients based upon *P* < 0.05. Those variables that were subjected to multifactor logistic regression analysis were then screened according to *P* < 0.2 to identify the variables obtained from logistic analysis. Based on the odds ratios (ORs) and 95% confidence intervals (95% CIs) for each variable, we calculated how much each characteristic was associated with infection risk. We then applied univariate logistic regression to determine which variables were independent risk factors.

### Identification of important characteristics

Intersection analysis was conducted to confirm the shared characteristics between the variables screened using the Support Vector Machine-Recursive Feature Elimination (SVM-RFE) model and those screened using logistic regression analysis (40–45). To determine the amount of information that these shared features can contain about the outcomes, PCA (principle component analysis) is applied to downscale them. In addition, neural network models with multilayer network structures are continuously used in several fields. Furthermore, these shared important features are also passed into the neural network model as input features to measure the classification performance of the variables under the neural network model.

### Construction of nomogram model

For the construction of infection prediction models, a multi-factor logistic regression model was used based on these important characteristics. By comparing the predicted probability values of the model to the actual results, the calibration curve further evaluates the accuracy of the model. A decision curve analysis (DCA) was used to evaluate the clinical safety of this clinical prediction model by measuring the yield at different prediction probability thresholds (46). The infection prediction model was then plotted using the “rms” package to obtain a clinical visualization tool to facilitate clinical translation.

### Statistical analysis

Software R (version 4.0.2) was used for all analyses and graphs. Training and evaluation of the individual prediction models were conducted by dividing the entire dataset into a training and a test set. The receiver operating characteristic curve (ROC) and the area under the curve (AUC) were used to evaluate the predictive performance of the model. PROC was used to plot ROC curves (47). Pearson's chi-square test or Fisher's exact test were used for the difference test analysis on the count data. The measurement data were analyzed using the Wilcoxon rank sum test or unpaired Student's *t-*test. We considered a significance level of *p* 0.05 to be statistically significant.

## Results

### Comparison of general characteristics and outcomes of patients between the two hydromorphone dosage groups

In the clinical study of 130 patients admitted to the ICU after surgery, 65 patients received an average daily hydromorphone dosage of more than 40 mg/mL and were included in the high hydromorphone dosage group; the remaining 65 patients were included in the low hydromorphone dosage group. An overview of the patients' baseline clinical information is given in Tables 1, 2. Hydromorphone dosage groups did not differ statistically in terms of biological sex, age, weight, height, BMI, hypertension, diabetes, APACHE II, and SOFA scores. However, the high hydromorphone dosage group had a high WBC count (*P* = 0.036) and a high infection risk (*P* = 0.014). This suggests that high hydromorphone dosages may increase the risk of infection.

**Table 1:** Comparison of baseline characteristics between the two hydromorphone consumption groups.

**Characteristic**		**Hydromorphone consumption**		***P*** **-value**
		**groups**		
		**High (*n =* 65)**	**Low** **(*n =* 65)**	
Biological	Female	20 (30.77)	26 (40.00)	0.271
sex (%)	Male	45 (69.23)	39 (60.00)	
Age, years		64.23 ± 8.97	61.18 ± 10.14	0.072
Weight, kg		65.98 ± 10.47	65.29 ± 10.96	0.713
Height, cm		170.26 ± 14.51	168.23 ± 13.77	0.415
BMI, kg/m^2^		22.74 ± 2.33	23.00 ± 1.98	0.491
Hypertension	No	60 (92.31)	54 (83.08)	0.109
(%)	Yes	5 (7.69)	11 (16.92)	
Diabetes (%)	No	58 (89.23)	51 (78.46)	0.095
	Yes	7 (10.77)	14 (21.54)	
APACHE_II		24.98 ± 5.75	24.09 ± 6.80	0.421
SOFA		4.15 ± 1.03	4.09 ± 1.03	0.734

*APACHE II, acute physiology and chronic health evaluation II; BMI, body mass index; SOFA, sepsis related organ failure assessment*.

**Table 2:** Comparison of clinical outcomes between the two hydromorphone consumption groups.

**Characteristic**		**Hydromorphone consumption groups**		***P***
		**High (*n =* 65)**	**Low** **(*n =* 65)**	
HR, min^−1^		95.77 ± 10.83	98.31 ± 11.38	0.195
MAP, mmHg		76.78 ± 7.47	76.89 ± 6.80	0.932
R, min^−1^		19.42 ± 3.61	20.52 ± 3.66	0.085
WBC, 10^9^/L		12.89 ± 5.11	11.23 ± 3.71	0.036*
PLT, 10^9^/L		231.58 ± 62.70	228.45 ± 43.61	0.741
CRP, mg/L		76.32 ± 31.25	74.38 ± 34.21	0.736
SpO_2_, %		96.43 ± 1.97	96.37 ± 2.43	0.874
ICU stay, day		2.25 ± 0.71	2.14 ± 0.61	0.354
LOH, day		9.66 ± 1.28	9.26 ± 1.12	0.06
Nausea	No	57 (87.69)	62 (95.38)	0.115
(%)	Yes	8 (12.31)	3 (4.62)	
Infection	No	47 (72.31)	58 (89.23)	0.014*
(%)	Yes	18 (27.69)	7 (10.77)	

CRP, C-reactive protein; HR, heart rate; ICU, intensive care unit; MAP, mean artery pressure; WBC, white blood cell; PLT, platelet; R, respiratory rate; *P < 0.05.

### Comparison of characteristics of infected and uninfected groups

Twenty-five patients developed postoperative infections, while the other 105 did not. Table 3 summarizes the clinical characteristics of the infected and uninfected patients. The occurrence of infection was associated with age (*P* = 0.006), BMI (*P* = 0.001), WBC count (*P* = 0.019), CRP (*P* = 0.038), hydromorphone dosage (*P* = 0.014), and biological sex (*P* = 0.024). Infected patients were older, had a lower BMI, higher WBC counts, higher CRP, and used more hydromorphone than non-infected patients. These results suggest that together with hydromorphone use, multiple clinical characteristics can be associated with the occurrence of postoperative infections in surgical patients.

**Table 3:** Clinical characteristics between the infected and uninfected groups.

**Characteristic**		**Infection**		***P***
		**No** **(*n* = 105)**	**Yes** **(*n* = 25)**	
Age, years		61.58 ± 9.55	67.44 ± 8.79	0.006**
Weight, kg		66.22 ± 10.32	63.20 ± 12.01	0.205
Height, cm		168.92 ± 13.33	170.60 ± 17.34	0.596
BMI, kg/m^2^		23.16 ± 2.14	21.64 ± 1.80	0.001**
APACHE_II		24.34 ± 6.30	25.36 ± 6.32	0.47
SOFA		4.10 ± 1.05	4.20 ± 0.96	0.679
HR, min^−1^		97.22 ± 11.48	96.28 ± 9.75	0.706
MAP, mmHg		76.75 ± 7.00	77.20 ± 7.74	0.779
R, min^−1^		19.96 ± 3.69	20.00 ± 3.65	0.963
WBC, 10^9^/L		11.61 ± 4.26	13.96 ± 5.18	0.019*
PLT, 10^9^/L		232.17 ± 50.04	220.96 ± 67.91	0.351
CRP, mg/L		72.46 ± 30.39	87.52 ± 39.23	0.038*
SpO_2_, %		96.40 ± 2.33	96.40 ± 1.61	1
Group (%)	High	47 (44.76)	18 (72.00)	0.014*
	Low	58 (55.24)	7 (28.00)	
Biological sex (%)	Female	42 (40.00)	4 (16.00)	0.024*
	Male	63 (60.00)	21 (84.00)	
Hypertension (%)	No	57 (87.69)	62 (95.38)	0.532
	Yes	8 (12.31)	3 (4.62)	
Diabetes (%)	No	47 (72.31)	58 (89.23)	0.218
	Yes	18 (27.69)	7 (10.77)	

APACHE II, acute physiology and chronic health evaluation II; BMI, body mass index; CRP, C-reactive protein; HR, heart rate; MAP, mean artery pressure; PLT, platelet; SOFA, sepsis related organ failure assessment; *P < 0.05, **P < 0.01.

### Ranking features based on machine learning

**Figure 1:**
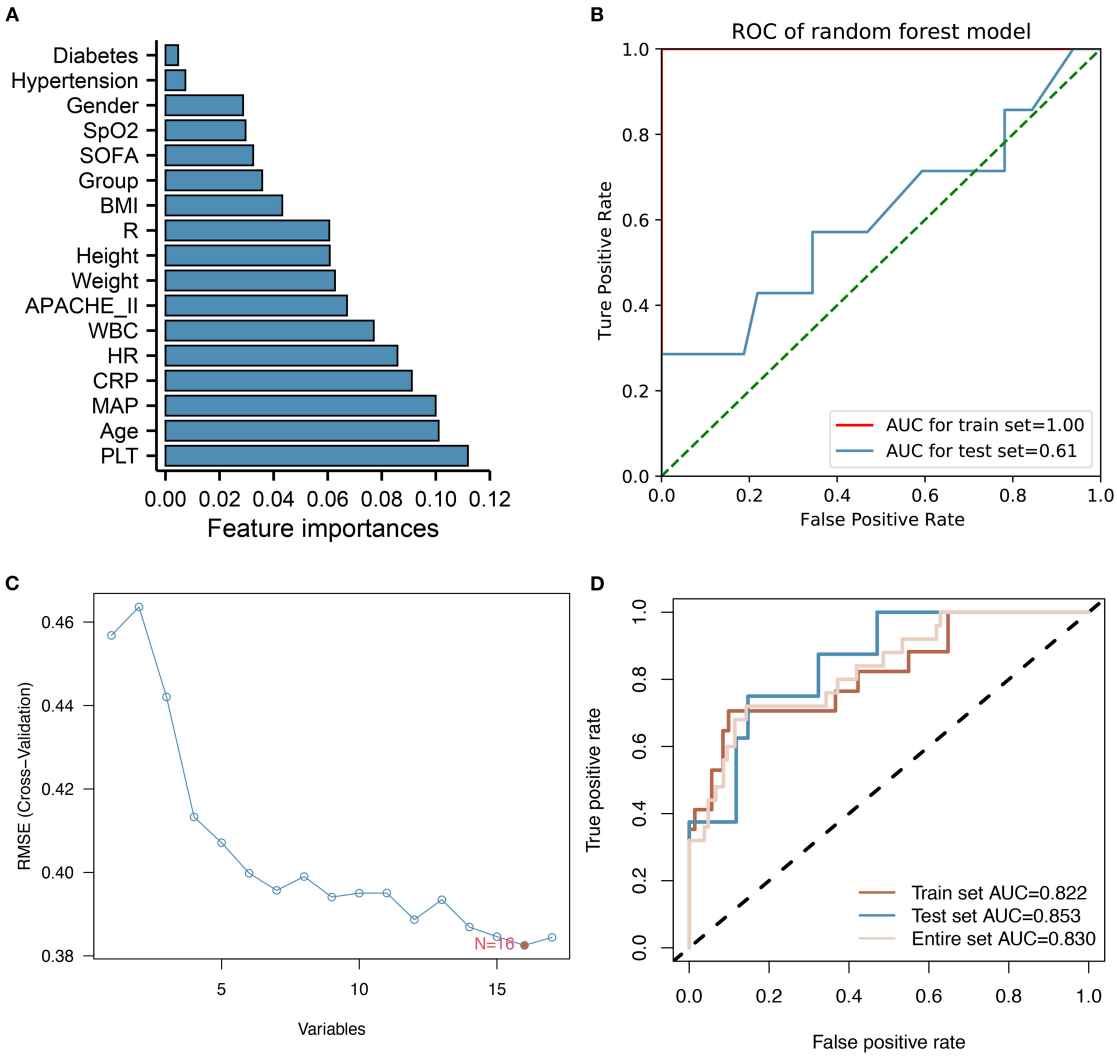
Feature ranking and filtering process for Random Forest and SVM-RFE models. **(A)** Bar chart showing a random forest model's importance ranking of each variable. PLT, age, MAP, CRP, and HR are the top five variables identified. **(B)** ROC curves showing the classification ability of the random forest model. **(C)** The feature screening process of SVM-RFE results in the model with the lowest RMSE when 16 variables are selected. **(D)** ROC curves showing training, test, and overall classification performances of the SVM model.

Based on a random forest model analysis, PLT, age, MAP, CRP, and HR were the top five clinical features associated with infection (Figure 1A). According to Figure 1B, the ROC curves for training and test sets had AUCs of 1.00 and 0.61, respectively, suggesting that the model was overfitted. After using the SVM-RFE method, we found that the model had the smallest Root Mean Square Error (RMSE) value when 16 variables were included (Figure 1C). AUCs of the SVM model based on these 16 variables were 0.822, 0.853, and 0.830, respectively, for the training, test, and overall datasets (Figure 1D). In the test set, SVM showed better classification ability than random forest model, according to the results of the appeal.

### Risk factors associated with infection

Risk factors associated with infection were identified using univariate and multifactorial logistic regression analyses. Univariate logistic regression analysis revealed an association between hydromorphone dosage, group (*P* = 0.018), age (*P* = 0.008), biological sex (*P* = 0.031), BMI (*P* = 0.002), WBC (*P* = 0.023), and CRP (*P* = 0.042). Furthermore, multifactorial logistic regression analysis revealed that age (*P* = 0.042), BMI (*P* = 0.005), and WBC (*P* = 0.031) were independent risk factors for infection (Table 4). Furthermore, a multifactorial logistic regression analysis with *P* < 0.2 as a filter identified Hydromorphone dosage group, age, biological sex, BMI, WBC count, and CRP as clinically important factors that contribute to infection risk.

**Table 4:** Univariate and multivariate logistics regression analysis.

**Variables**	**Univariate**			**Multivariate**	
	**OR (95% CI)**	***P*** **value**	**β**	**OR (95% CI)**	***P*** ** value**
Group (High vs. Low)	3.17 (1.27–8.76)	0.018*	0.83	2.3 (0.79–7.32)	0.137
Age	1.07 (1.02–1.12)	0.008**	0.06	1.07 (1.01–1.13)	0.02*
Biological sex (Male vs. Female)	3.5 (1.23–12.64)	0.031*	1.1	3.02 (0.9–12.59)	0.094
Weight	0.97 (0.93–1.01)	0.205			
Height	1.01 (0.98–1.04)	0.593			
BMI	0.7 (0.55–0.87)	0.002**	−0.38	0.68 (0.51–0.88)	0.005**
Hypertension (Yes/No)	1.48 (0.38–4.73)	0.534			
Diabetes (Yes/No)	0.39 (0.06–1.49)	0.232			
APACHE_II	1.03 (0.96–1.1)	0.467			
SOFA	1.1 (0.71–1.68)	0.676			
HR	0.99 (0.95–1.03)	0.704			
MAP	1.01 (0.95–1.07)	0.777			
R	1 (0.89–1.13)	0.963			
WBC	1.12 (1.02–1.24)	0.023*	0.14	1.15 (1.02–1.31)	0.031*
PLT	1 (0.99–1)	0.349			
CRP	1.01 (1–1.03)	0.042*	0.01	1.01 (1–1.03)	0.094
SpO2	1 (0.82–1.23)	1			

APACHE II, acute physiology and chronic health evaluation II; BMI, body mass index; CRP, C-reactive protein; HR, heart rate; MAP, mean artery pressure; PLT, platelet; R, respiration; SOFA, sepsis related organ failure assessment; SpO2, Saturation of Pulse Oxygen; WBC, white blood cell; *P < 0.05, **P < 0.01.

### Variables associated with infection outcome identification

**Figure 2:**
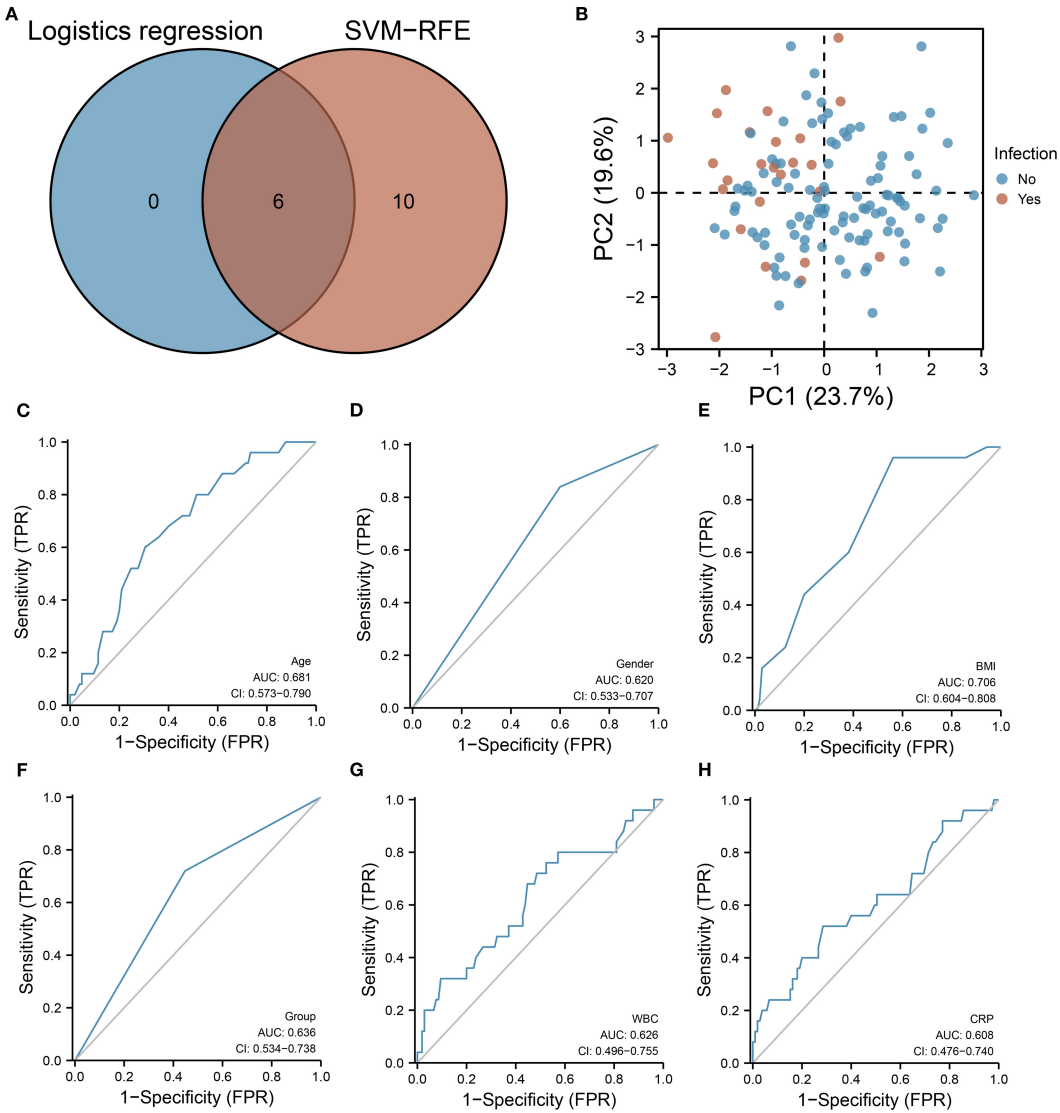
SVM-RFE as well as logistic regression models are used for screening important clinical features. **(A)** Venn diagram showing six features associated with infection prediction. **(B)** PCA showing that based on these six characteristics, a better distinction can be made between infected and uninfected patients. **(C**–**H)** ROC curves showing the predictive performance of **(C)** age, **(D)** biological sex, **(E)** BMI, **(F)** hydromorphone concentration grouping, **(G)** WBC, and **(H)** CRP on infection.

According to the intersection analysis of SVM-RFE and logistic regression analysis, six variables were coinciding with the SVM-RFE and logistic regression analysis, including hydromorphone dosage group, age, biological sex, BMI, WBC count, and CRP (Figure 2A). These six variables have sufficient information to predict infection occurrence according to principal component analysis (PCA) (Figure 2B). According to ROC curve analysis, age (AUC = 0.681), biological sex (AUC = 0.620), BMI (AUC = 0.706), dosage group (AUC = 0.636), WBC count (AUC = 0.626), and CRP (AUC = 0.608) were all predictive of infection (Figures 2C–H). Supplementary figure 1 illustrates the training process of the neural network model based on these infection-related variables. In both the training and validation sets, the neural network model accurately predicted infection. However, the neural network model only performed 0.539 accurately in the test set. It is possible that this deficiency is due to the small number of patients included in this study.

### Construction and evaluation of the nomogram prediction model

**Figure 3:**
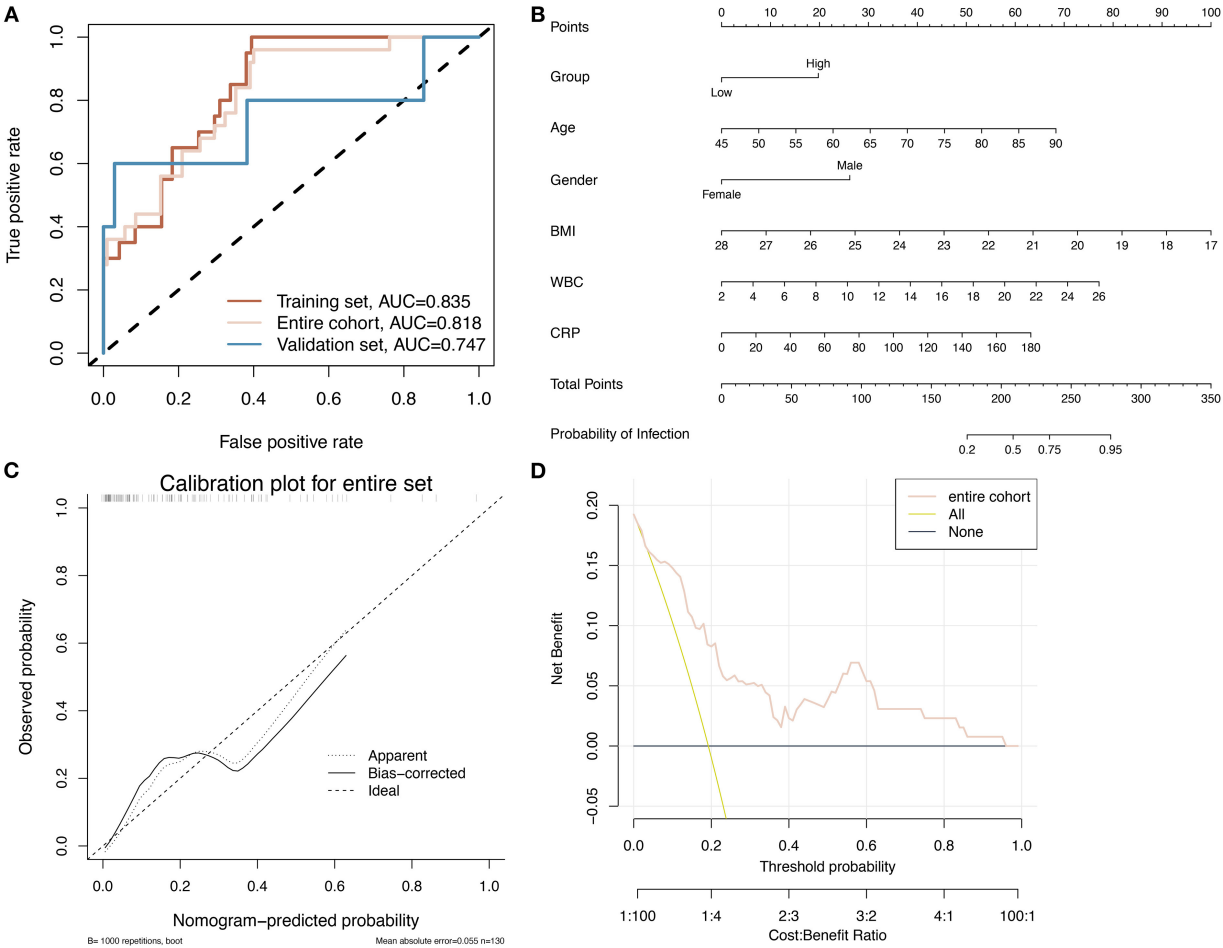
A nomogram based on six factors is constructed and its accuracy is assessed. **(A)** The ROC curves for the logistic regression model constructed based on the six identified clinical factors for infection in training, validation, and overall pairwise column sets demonstrate better classification performance in all three datasets. **(B)** The nomogram was constructed using a logistic regression model. **(C)** Calibration plot showing the predicted values of the model are roughly consistent with the true labels, indicating that the model is reasonably accurate. **(D)** Clinical decision curve showing the prediction results in an overall pairwise column.

Based on the six identified clinical variables, a multifactorial logistic regression algorithm was applied to construct a prediction model to detect the occurrence of infection in relation to the clinical use of hydromorphone. Table 4 shows the coefficients of the multifactor logistic regression model based on these six clinical characteristic variables. In the training group, validation group, and overall pairwise column, respectively, the ROC curves showed an AUC of 0.835, 0.818, and 0.747, indicating the model has good predictive ability (Figure 3A). Based on the multifactor logistic regression model, a nomogram was constructed (Figure 3B) to facilitate its clinical application. The correction curves show that the predicted values of the infection prediction model and the true label are in general agreement, further supporting the good predictive power of the model (Figure 3C). Clinical decision curves show that the predictions from the prediction model have clinical value for patients (Figure 3D). Projecting the basic characteristics of the patients and hydromorphone use onto this nomogram allows for the easy calculation of the probability of infection risk, thus guiding hydromorphone use and patient testing in the clinical setting.

**Figure 4:**
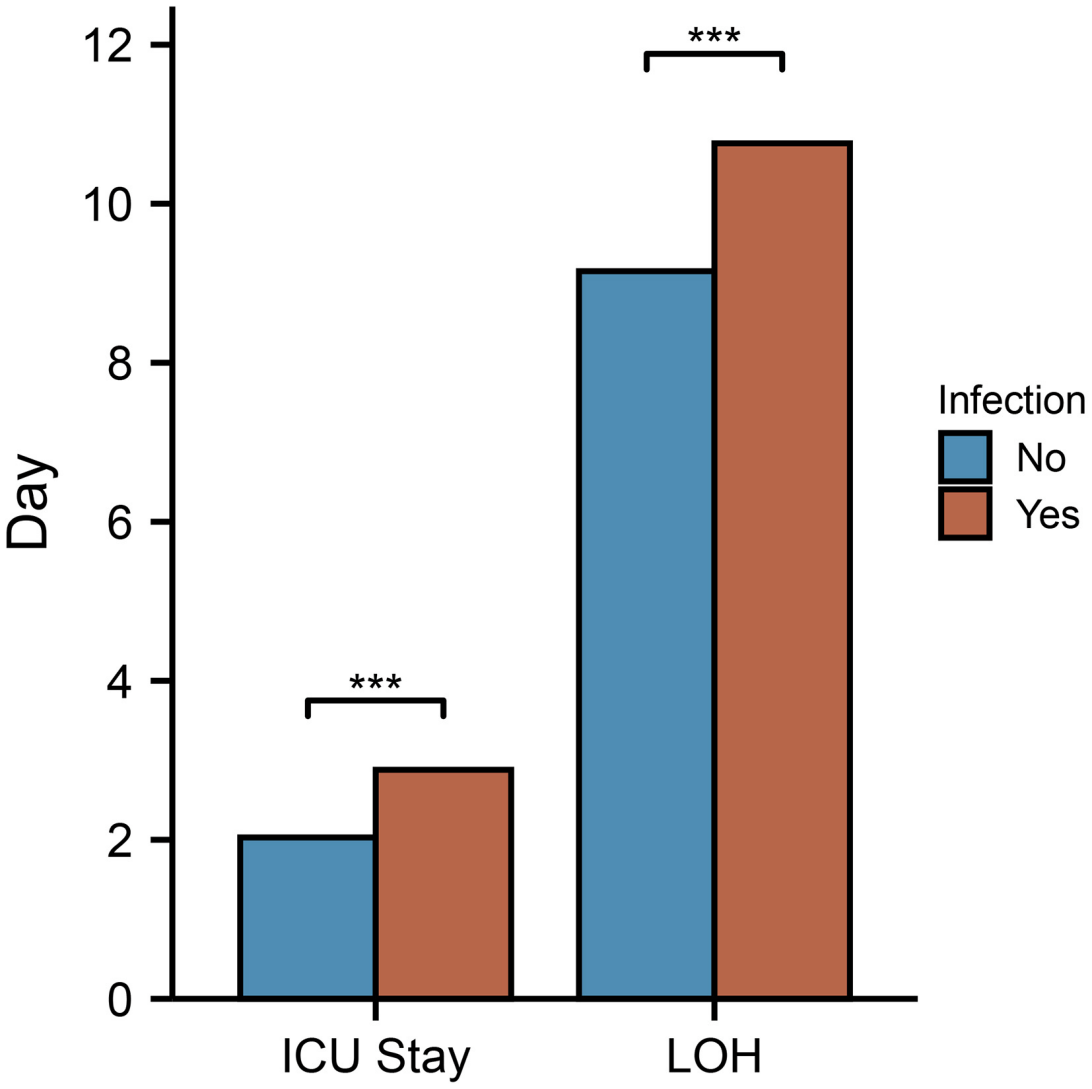
Compared between infected and non-infected patients, duration of ICU stay and hospitalization. In the infected group, the hospital stay and ICU stay were longer than in the non-infected group.

In addition, infected patients spent more time in the intensive care unit and hospital overall than their non-infected counterparts (Figure 4). Infections in intensive care units can prolong the stay in the unit, resulting in increased healthcare costs.

## Discussion

A machine learning approach was used to predict the risk of infection among non-mechanically ventilated ICU patients receiving hydromorphone analgesia after surgical procedures. According to the study, different factors are associated with the occurrence of postoperative infections in surgical patients based on a cohort study. We identified significant risk factors affecting the development of postoperative infections in patients based on these data, including hydromorphone dosage, age, biological sex, BMI, WBC count, and CRP. Logistic regression was used to construct a predictive model for detecting infection. To assist physicians in assessing the risk of postoperative infections in surgical patients, a nomogram was created based on this model.

In postoperative surgical patients, infections are common adverse effects of opioid analgesics, which can reduce their quality of life, and prolong their length of stay (48–51). A high hydromorphone dosage was associated with a higher incidence of postoperative infections in this study. It has been shown that patients on hydromorphone have less than normal immune system defenses, which may allow bacteria and viruses to infiltrate the body and multiply (20, 52). It has also been shown that hydromorphone, as an opioid analgesic, can cause adverse effects, such as excessive sedation and respiratory depression, pulmonary atelectasis, and infection (53–55). Additionally, hydromorphone promotes bacterial translocation by breaking endothelial tight junctions *via* the Toll-like receptor 2 (20). Opioids increase intestinal bacterial translocation, dysregulated immune responses, and intestinal barrier permeability, thereby increasing the risk of intestinal infections (56). We also found that the WBC count and CRP after 12 h of analgesia were higher in the high hydromorphone dosage group than in the low hydromorphone dosage group. Clinical parameters such as these have been shown to be risk factors for postoperative infection in this study. Infections are more likely to occur in the high hydromorphone dosage group, according to these findings.

Among patients admitted to the ICU after surgery and receiving analgesic treatment, old age was an independent risk factor for infection development. There may be an important correlation between this finding and low immunity in the elderly (57). Similarly, low BMI was identified as an independent risk factor for infection, possibly due to findings that low BMI correlates with worse nutritional status, postoperative recovery, and greater susceptibility to infection (58–60). Furthermore, males had a higher proportion of infections in postoperative ICU admissions than females. Different sex hormones induce different gene expression and immune responses in males and females, which may contribute to different susceptibility to infection (61). Estradiol appears to confer protective immunity, while progesterone and testosterone suppress anti-infection responses (62). Occupational differences and lifestyle differences may also play a role. Therefore, post-analgesic infections should be closely monitored in patients over the age of 65, those with low body mass indexes, and those admitted to the ICU after surgery.

According to our analysis, patients in the infected group spend more time in the ICU and in the hospital, resulting in higher costs and more resource utilization. Infection is a common adverse effect of opioid analgesics in postoperative surgical patients (48, 49). Postoperative infection can negatively impact the patient's prognosis, which negatively impacts their recovery (50, 51). Patients will benefit medically and economically from the early detection and timely treatment of infections after surgery in the ICU.

Despite the higher risk of infection associated with high doses of hydromorphone, postoperative pain control can improve patient recovery. There is evidence that timely and effective relief of postoperative pain enhances recovery, leads to fewer complications, and shortens hospital stays (63). Hydromorphone is commonly used as a bout of pain medication; however, its use also introduces a range of side effects. In order to identify infection-related factors, we used logistic regression to construct infection prediction models and developed an easy-to-use clinical infection prediction nomogram. The tool will help physicians evaluate the risk of infection in surgical patients using hydromorphone for analgesia promptly and may enable early medical intervention, as required by precision medicine. Pain control contributes to the recovery of surgical patients, and timely detection and reduction of analgesic medication use can reduce adverse effects and decrease ICU and hospital stays. A patient-centered big medical data set should be constructed in future studies by collecting all manner of basic patient information, treatment information, and outcomes indicators with the aid of various machine learning predictive models. It is important to design prospective cohort studies to further validate this model, as well as to expand the study population to include different types of surgical and postoperative infections in order to achieve precision medicine.

## Conclusion

This study explores and clarifies hydromorphone's efficacy and safety in the ICU. Hydromorphone dosage, age, biological sex, BMI, WBC count, and CRP have been found to be significant risk factors for developing postoperative infections in non-mechanically ventilated patients in the ICU after surgery. Based on these six clinical variables, infection prediction models have good predictive power and can be used to guide hydromorphone use more safely.

## Data Availability Statement

The original contributions presented in the study are included in the article/Supplementary material, further inquiries can be directed to the corresponding author.

## Ethics Statement

The studies involving human participants were reviewed and approved by the Ethics Committee of Shanxi Bethune Hospital. The patients/participants provided their written informed consent to participate in this study.

## Author contributions

YD and WW: conceptualization and validation. YD: methodology. HS: software. YD and XY: data curation. YD, HS, WW, and XY: writing—original draft preparation. WW: writing—review and editing. All authors contributed to the article and approved the submitted version.

## Conflict of interest

The authors declare that the research was conducted in the absence of any commercial or financial relationships that could be construed as a potential conflict of interest.

## Publisher's note

All claims expressed in this article are solely those of the authors and do not necessarily represent those of their affiliated organizations, or those of the publisher, the editors and the reviewers. Any product that may be evaluated in this article, or claim that may be made by its manufacturer, is not guaranteed or endorsed by the publisher.

## Supplementary Material

Supplementary Figure 1 The training process for the neural network prediction model based on the six identified clinical features. The line graphs illustrate the **(A)** loss of training and validation, and **(B)** changes in AUC values for the prediction models in the training and validation sets during training.
