# Supplementary figures and images for: Machine learning for infection risk prediction in postoperative patients with non-mechanical ventilation and intravenous neurotargeted drugs
Source: Front Neurol. 2022 Aug 1;13:942023. doi: 10.3389/fneur.2022.942023 (PMC9376287; doi:10.3389/fneur.2022.942023)

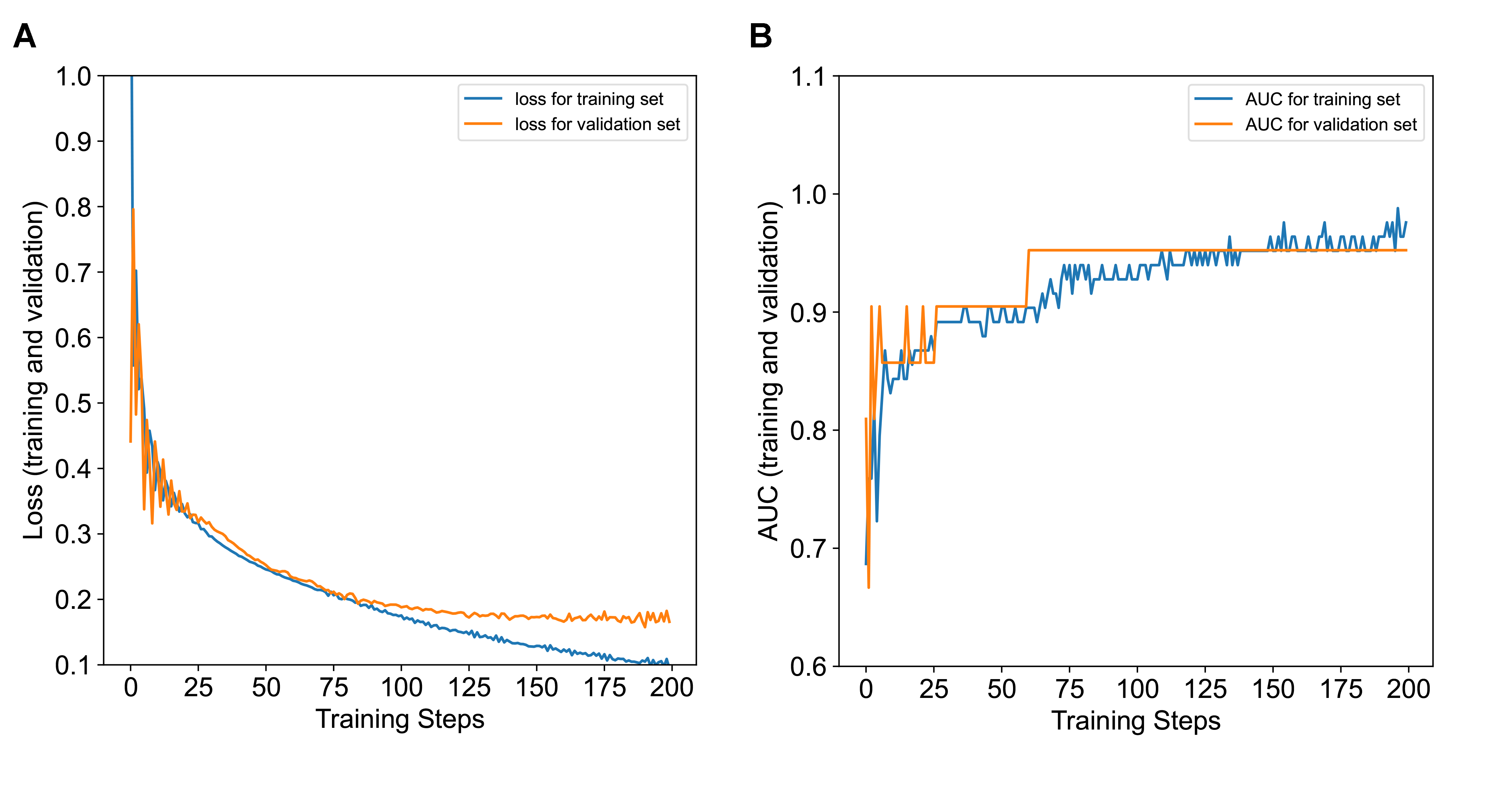

Supplement: Supplementary Figure 1 — The training process for the neural network prediction model based on the six identified clinical features. The line graphs illustrate the (A) loss of training and validation, and (B) changes in AUC values for the prediction models in the training and validation sets during training. [file Image_1.TIF]
